# Variant of Uncertain Significance Patterns among Patients with Early-Onset Colorectal Cancer

**DOI:** 10.1158/2767-9764.CRC-24-0368

**Published:** 2025-02-13

**Authors:** Rachel A. Francis, Sean Tavtigian, Carolyn Horton, Andreana N. Holowatyj

**Affiliations:** 1 Department of Medicine, Vanderbilt University Medical Center, Nashville, Tennessee.; 2 Department of Oncological Sciences, University of Utah School of Medicine, Salt Lake City, Utah.; 3 Department of Clinical Diagnostics, Ambry Genetics, Aliso Viejo, California.; 4 Department of Population Health Sciences, University of Utah School of Medicine, Salt Lake City, Utah.; 5 Vanderbilt-Ingram Cancer Center, Nashville, Tennessee.; 6 Vanderbilt University School of Medicine, Nashville, Tennessee.

## Abstract

The increasing burden of colorectal cancer among adults younger than age 50 years (early-onset colorectal cancer) and new National Comprehensive Cancer Network guidelines recommending universal genetic testing in early-onset colorectal cancer emphasize the need for accurate and timely variant classification in clinical care. In this study, we investigated germline variant of uncertain significance (VUS) patterns among diverse individuals with early-onset colorectal cancer. A total of 3,980 individuals ages 15 to 49 years when diagnosed with a first primary colorectal cancer, including 1,001 cases identifying as non-White, who underwent genetic testing for 14 colorectal cancer susceptibility genes performed by a clinical testing laboratory were included. A five-tier classification system was applied to all alterations to classify VUSs and was updated in March 2024. Disparities in variant reclassification rates emerged by self-identified race/ethnicity (*P* < 0.0001). A total of 4.8% of Ashkenazi Jewish, 18.2% of Asian, 12.2% of Black, 7.6% of Hispanic, and 6.7% of White individuals had at least one reclassified VUS. After reclassification, 356 individuals (8.9%) presented with 1+ VUSs. VUSs significantly varied by self-identified race/ethnicity (*P* = 0.008). Young individuals identifying as Black and Hispanic had significantly higher odds of presenting with a VUS compared with those identifying as White in multivariable logistic regression models. Individuals who identified as Black and Asian were significantly more likely to present with a VUS in *PMS2* (OR = 3.59; 95% confidence interval, 1.73–7.48; *P* = 0.0006) and *MSH2* (OR = 3.14; 95% confidence interval, 1.17–8.45; *P* = 0.02), respectively, compared with those identifying as White. These findings define unique VUS patterns in early-onset colorectal cancer by race and ethnicity, pointing to distinct germline variant spectra and to the potential for the discovery of novel ancestry-specific variants associated with early-onset colorectal cancer that will guide efforts to reduce clinical uncertainty and improve equitable care.

**Significance:**

Among individuals with early-onset colorectal cancer, germline VUS reclassification as well as rates of VUSs in cancer susceptibility genes differed by self-identified race/ethnicity. These findings point to the importance of VUS reclassification as this may alter clinical management and to distinct germline variant spectra among diverse patients with early-onset colorectal cancer.

## Introduction

With rising rates of colorectal cancer incidence among individuals younger than 50 years of age (early-onset colorectal cancer), it is now estimated that one in every 239 males and one in every 265 females will be diagnosed with this cancer type before 50 years of age in the United States (1). Based on current trends, early-onset colorectal cancer incidence rates are expected to continue climbing. It is estimated that colon cancer–specific rates will increase by 90% and 27.7% in 2030 for individuals ages 20 to 34 years and those ages 35 to 49 years, respectively (2). For rectal cancers, these rates are expected to increase even higher, by 46.0% and 124.2%, respectively. Yet, it remains unclear what the etiologies underpinning this colorectal cancer epidemic are in young individuals.

An established predisposing factor to early-onset colorectal cancer development is the inheritance of genetic variants in cancer risk–related genes. Prior studies have estimated the prevalence of germline genetic features in early-onset colorectal cancer (3–7), finding that approximately one in every six patients harbors a deleterious sequence variation in a known cancer susceptibility gene. The historical guidelines for selection of young patients with colorectal cancer to undergo germline genetic testing have largely relied upon family cancer history and tumor-based criteria. However, recent discoveries that universal genetic testing can detect more clinically actionable germline variants over this guideline-based approach (8, 9) prompted the National Comprehensive Cancer Network to update their 2023 clinical practice guidelines with the recommendation that *all* individuals diagnosed with early-onset colorectal cancer should undergo germline multigene panel testing (10). Among the plethora of advantages to this more sensitive approach of identifying individuals with Lynch syndrome and those who harbor other deleterious variants in cancer susceptibility genes are the immediate opportunities for informing surgical and treatment decision-making, eliminating the dependence on knowledge of family cancer history (11, 12), screening and surveillance recommendations, and directing cascade family variant testing among at-risk family members.

It is important to consider that the successful implementation of this new universal germline genetic testing strategy in early-onset colorectal cancer critically relies upon a sufficient capacity to deliver pretest and posttest counseling to all individuals—those with deleterious variants, those with variants of uncertain significance (VUSs), and at least a subset of those with negative results. Although gene-based clinical management recommendations are limited to deleterious [pathogenic variant (PV) and likely pathogenic variant (VLP)] variants across cancer susceptibility genes, there is no current method to predict which VUSs will be upgraded to a PV/VLP with clinical accuracy over time. This clinical uncertainty, together with the expectation that the number of individuals with VUSs is expected to increase in the future as more individuals diagnosed with early-onset colorectal cancer are tested, the testing gene panel size is expanded, and the access to genetic testing is increased, stresses the importance of accurate and timely variant classification. Overall, the low proportion of variants that are reclassified from a VUS over time (13) and the downstream clinical implications illuminate the urgent need for sufficient information and optimized tools to validate pathogenicity of variants *across diverse population groups* in support of high-quality, long-term clinical care to each young patient diagnosed with colorectal cancer.

The study of VUSs among diverse individuals ages 15 to 49 years when diagnosed with a first primary colorectal cancer offers a unique opportunity to inform which patients may harbor a higher propensity for clinical uncertainty/VUSs. Such efforts are an integral first step toward mitigating these key gaps for optimal and equitable clinical care management and reducing the genetic risk of early-onset colorectal cancer. Here, we comprehensively characterized VUS patterns among 3,980 individuals with a documented first primary early-onset colorectal cancer [we have previously described the cohort in Seagle and colleagues (4)], which includes 1,001 individuals identifying as non-White (25.2%), who uniformly underwent germline genetic testing for *APC*, *BMPR1A*, *CDH1*, *CHEK2*, *EPCAM*, *MLH1*, *MSH2*, *MSH6*, *MUTYH*, *PMS2*, *PTEN*, *SMAD4*, *STK11*, and *TP53* by a nationwide clinical testing laboratory.

## Materials and Methods

A total of 3,980 individuals diagnosed with a first primary colorectal cancer between ages 15 and 49 years (regardless of a family history of colorectal cancer) who self-identified as Ashkenazi Jewish, Asian, Black, Hispanic, or White and underwent comprehensive germline multigene panel testing for 14 colorectal cancer susceptibility genes at a clinical testing laboratory between March 2012 and December 2016 were included in this study and are previously described in Seagle and colleagues (4). Because the data presented herein were deidentified and aggregated, the WGC Institutional Review Board (formerly the Western Institutional Review Board) determined this study to be exempt from the Office for Human Research Protections Regulations for the Protection of Human Subjects (45 CFR 46).

Clinical and demographic information was completed by the ordering provider on test requisition forms, visit notes, pedigrees, and pathology reports. High data accuracy on test requisition forms for cancer diagnosis among probands has been confirmed in prior studies (14). All individuals had germline genetic testing results from a pan-cancer or targeted colorectal cancer clinical panel (CancerNext and ColoNext, respectively) for *APC* (NM_000038.6), *BMPR1A* (NM_004329.2), *CDH1* (NM_004360.5), *CHEK2* (NM_007194.4), *EPCAM* (NM_002354.2), *MLH1* (NM_000249.3), *MSH2* (NM_000251.2), *MSH6* (NM_000179.2), *MUTYH* (NM_001128425.1), *PMS2* (NM_000535.7), *PTEN* (NM_000314.7), *SMAD4* (NM_005359.5), *STK11* (NM_000455.4), and *TP53* (NM_000546.4). Genetic analysis consisted of full-gene sequencing of coding regions plus five base pairs into exon/intron boundaries and gross deletion/duplication testing, except for *EPCAM* (only gross deletion/duplication testing; ref. 15). As previously described (16), a five-tier classification system was applied to all genetic alterations to classify PV/VLPs and VUSs (4). Variant classification was updated in March 2024, in which upgraded variants were also reclassified to a PV/VLP in this present study.

Demographic and clinical features of the study population by VUS status were summarized by frequency. Differences in individual and genetic features by VUS status were compared using χ^2^ tests and *t* tests for categorical and continuous variables, respectively. Overall and individual gene VUS patterns by self-identified race and ethnicity were quantified using multivariable logistic regression models adjusted for patient sex at birth (male/female), age at colorectal cancer diagnosis (years), prior cancer history (yes/no), and status of a clinically actionable PV/VLP (yes/no). Data were analyzed using SAS v9.4 software (SAS Institute; RRID: SCR_008567). All tests were two-sided with *P* < 0.05 considered to be statistically significant.

### Data availability

The clinical testing data generated in this study are not publicly available because of patient privacy requirements and were made available for this secondary analysis from Ambry Genetics (ClinVar Organization ID: 61756). The data are available from the authors with the permission of Ambry Genetics.

## Results

**Figure 1:**
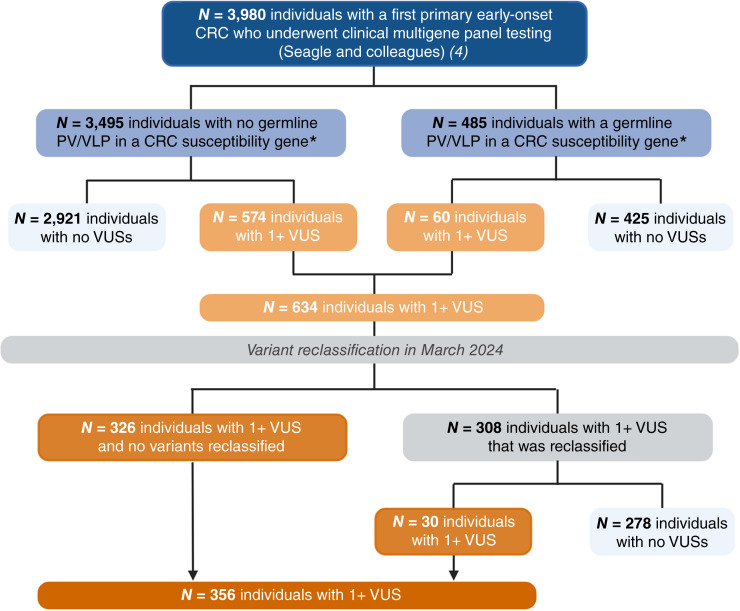
Composition of the study population by germline VUS status. *Colorectal cancer (CRC) susceptibility genes include *APC*, *BMPR1A*, *CDH1*, *CHEK2*, *EPCAM*, *MLH1*, *MSH2*, *MSH6*, *MUTYH*, *PMS2*, *PTEN*, *SMAD4*, *STK11*, and *TP53*.

**Figure 2:**
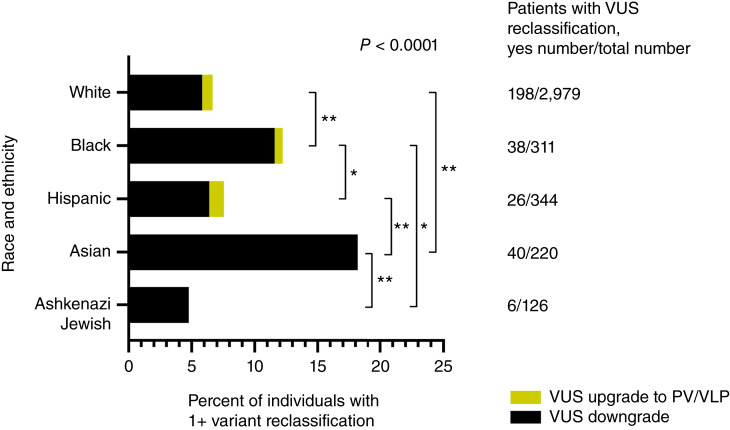
Variant reclassification by race and ethnicity among 308 individuals with early-onset colorectal cancer. All patients with a VUS upgraded to a PV/VLP also had at least 1 downgraded VUS. Pairwise: *, *P* < 0.05; **, *P* ≤ 0.01.

Among the 3,980 diverse individuals diagnosed with early-onset colorectal cancer in our cohort, a total of 720 VUSs were originally identified in 634 patients (4). Variant reclassification was performed in March 2024, in which a total of 332 VUSs were reclassified in 308 patients (Fig. 1). Among these reclassified VUSs, 27 different variants were upgraded from a VUS to a PV/VLP in 30 patients—26 of whom previously had no germline PV/VLP in any of the 14 colorectal cancer susceptibility genes evaluated. Reclassification rates by self-identified race and ethnicity among the 308 individuals are presented in Fig. 2. Strikingly, disparities in VUS reclassification emerged by self-identified race and ethnicity (*P* < 0.0001)—a total of 4.8% of individuals identifying as Ashkenazi Jewish, 18.2% as Asian, 12.2% as Black, 7.6% as Hispanic, and 6.7% as White had at least one reclassified VUS.

**Figure 3:**
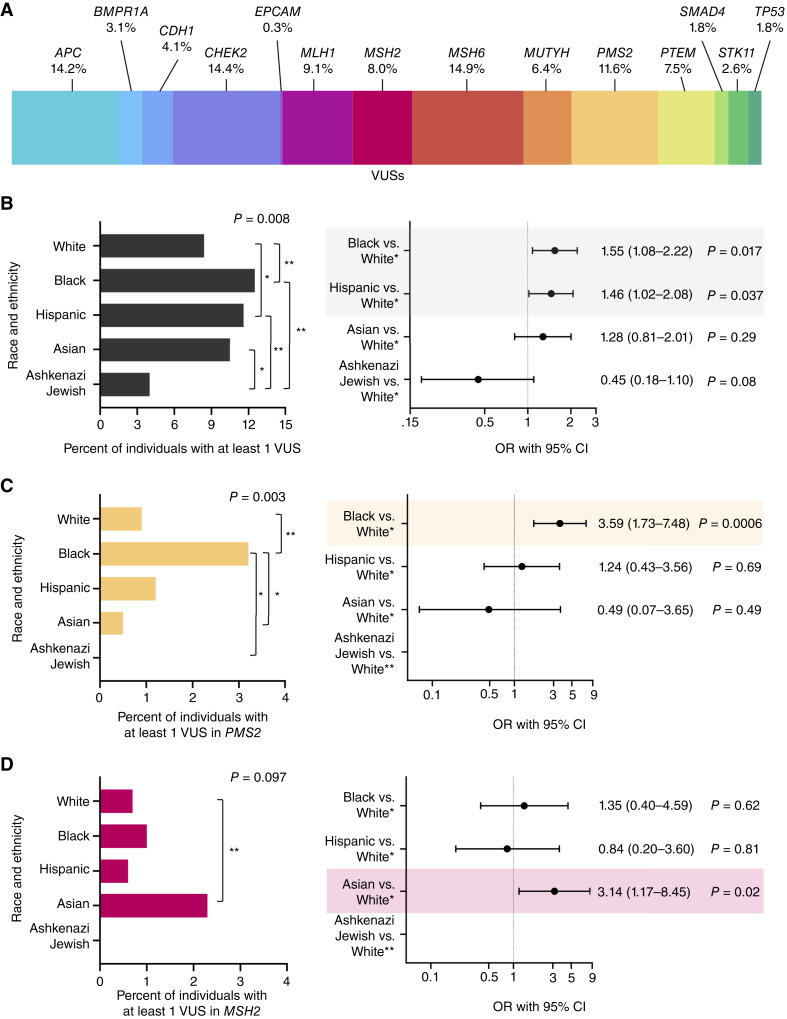
Distinct patterns of VUSs among individuals with early-onset colorectal cancer. **A,** Spectrum of 388 VUSs identified among 356 individuals in this cohort. **B,** (Left) Prevalence of VUSs by race and ethnicity. Pairwise: *, *P* < 0.05; **, *P* ≤ 0.01. Right, Forest plot depicting odds of a VUS in a cancer susceptibility gene in adjusted models. Cancer susceptibility genes include *APC*, *BMPR1A*, *CDH1*, *CHEK2*, *EPCAM*, *MLH1*, *MSH2*, *MSH6*, *MUTYH*, *PMS2*, *PTEN*, *SMAD4*, *STK11*, and *TP53*. *Models are adjusted for age at early-onset colorectal cancer diagnosis, sex, prior cancer history, and status of a detected clinically actionable variant. Dot/circle represents the OR, and bars represent the 95% CI. Bars shaded in color indicate *P* < 0.05. **C,** (Left) Prevalence of VUSs in *PMS2* by race and ethnicity. Pairwise: *, *P* < 0.05; **, *P* ≤ 0.01. Right, Forest plot depicting odds of a VUS in *PMS2*. *Models are adjusted for age at early-onset colorectal cancer diagnosis, sex, prior cancer history, and status of a detected clinically actionable variant. Dot/circle represents the OR, and bars represent the 95% CI. Bars shaded in color indicate *P* < 0.05. **There were no individuals who identified as Ashkenazi Jewish with a VUS in *PMS2* in this cohort. **D,** (Left) Prevalence of VUSs in *MSH2* by race and ethnicity. Pairwise: *, *P* < 0.05; **, *P* ≤ 0.01. Right, Forest plot depicting odds of a VUS in *MSH2*. Bars shaded in color indicate *P* < 0.05. *Models are adjusted for age at early-onset colorectal cancer diagnosis, sex, prior cancer history, and status of a detected clinically actionable variant. Dot/circle represents the OR, and bars represent the 95% CI. Bars shaded in color indicate *P* < 0.05. **There were no individuals who identified as Ashkenazi Jewish with a VUS in *MSH2* in this cohort.

After variant reclassification, a total of 388 VUSs remained among 356 individuals (8.9%; Table 1). The prevalence and spectrum of these VUSs are presented in Fig. 3A and Supplementary Table S1. No differences in patient sex, age at early-onset colorectal cancer diagnosis, prior cancer history, or the presence of a clinically actionable variant were observed by VUS status (all *P* ≥ 0.05; Table 1). In contrast, the proportion of individuals with early-onset colorectal cancer who had a VUS significantly varied by self-identified race and ethnicity (*P* = 0.008)—from 4.0% of individuals who identified as Ashkenazi Jewish, 8.4% of individuals who identified as White, 10.5% of individuals who identified as Asian, and 11.6% of individuals who identified as Hispanic to 12.5% of individuals who identified as Black presenting with at least one VUS in a colorectal cancer susceptibility gene (Fig. 3B; Table 1; Supplementary Tables S2 and S3). Overall, the odds of carrying a VUS were 55% and 46% higher among individuals who identified as Black and Hispanic, respectively, compared with those who identified as White—after adjusting for age at early-onset colorectal cancer diagnosis, sex, prior cancer history, and status of a clinically actionable variant [Black vs. White: OR, 1.55; 95% confidence interval (CI), 1.08–1.22; *P* = 0.017; Hispanic vs. White: OR, 1.46; 95% CI, 1.02–2.08; *P* = 0.037; Fig. 3B; Supplementary Table S4].

**Table 1:** Summary of clinicodemographic features by VUS status among individuals with early-onset colorectal cancer

	VUS status^a^				
	No VUS detected		One or more VUSs detected		*P*
Characteristic	Number	%	Number	%	
Total	3,624	91.1	356	8.9	
Self-reported race and ethnicity					0.008
White	2,730	91.6	249	8.4	
Black	272	87.5	39	12.5	
Hispanic	304	88.4	40	11.6	
Asian	197	89.6	23	10.5	
Ashkenazi Jewish	121	96.0	5	4.0	
Sex					0.09
Male	1,564	92.0	137	8.1	
Female	2,060	90.4	219	9.6	
Age at CRC diagnosis, years					0.9
15–29	352	91.2	34	8.8	
30–39	1,219	90.8	124	9.2	
40–49	2,053	91.2	198	8.8	
Mean (SD), years	39.6	(7.0)	39.6	(6.8)	0.95
Prior cancer history					0.66
No	3,440	91.1	336	8.9	
Yes	184	90.2	20	9.8	
Clinically actionable variant (PV and/or VLP) detected^a^					0.05
No	3,147	90.7	322	9.3	
Yes	477	93.4	34	6.7	

Percent values are calculated as row percentages.

Abbreviation: CRC, colorectal cancer.

a Variant classification as of March 2024. Genes include *APC*, *BMPR1A*, *CDH1*, *CHEK2*, *EPCAM*, *MLH1*, *MSH2*, *MSH6*, *MUTYH*, *PMS2*, *PTEN*, *SMAD4*, *STK11*, and *TP53*.

To further investigate these observed racial and ethnic differences in VUSs, we also characterized VUS patterns by individual colorectal cancer susceptibility genes (Table 2). Approximately one in every 10 VUSs within this early-onset colorectal cancer cohort was detected in *PMS2*—with the prevalence of *PMS2* VUSs statistically significantly differing by race and ethnicity (*P* = 0.003; Fig. 3C). Specifically, VUSs in *PMS2* varied between individuals who identified as Black and those who identified as Ashkenazi Jewish, Asian, and White (all pairwise *P* < 0.05; Supplementary Table S5). Among individuals who identified as Black, all cases with a *PMS2* VUS had only one VUS in *PMS2* (Table 3). Moreover, 80% of these cases (*n* = 8 of 10) did not harbor a clinically actionable PV/VLP in any colorectal cancer susceptibility genes evaluated, and 90% had no prior cancer history. For *PMS2*, these racial and ethnic differences persisted in the multivariable models as individuals who identified as Black were 3.59-fold more likely to present with a VUS in *PMS2* compared with those who identified as White (OR, 3.59; 95% CI, 1.73–7.48; *P* = 0.0006; Fig. 3C; Supplementary Table S6).

**Table 2:** Prevalence of VUSs in 14 colorectal cancer susceptibility genes identified among patients with early-onset colorectal cancer by race and ethnicity

		Race and ethnicity										
	Study Population^a^	White		Black		Hispanic		Asian		Ashkenazi Jewish		*P*
	Number	Number	%	Number	%	Number	%	Number	%	Number	%	
Total	3,980	2,979		311		344		220		126		
Gene												
*APC*	55	41	1.4	5	1.6	7	2.0	2	0.9	—	—	0.51
*BMPR1A*	12	7	0.2	1	0.3	3	0.9	1	0.5	—	—	0.32
*CDH1*	16	9	0.3	1	0.3	4	1.2	1	0.5	1	0.8	0.18
*CHEK2*	54	40	1.3	2	0.6	9	2.6	2	0.9	1	0.8	0.21
*EPCAM*	1	1	0.03	—	—	—	—	—	—	—	—	0.99
*MLH1*	36	26	0.9	4	1.3	4	1.2	2	0.0	—	—	0.75
*MSH2*	31	21	0.7	3	1.0	2	0.6	5	2.3	—	—	0.097
*MSH6*	57	39	1.3	5	1.6	7	2.0	5	2.3	1	0.8	0.60
*MUTYH*	23	16	0.5	2	0.6	—	—	3	1.4	2	1.6	0.15
*PMS2*	43	28	0.9	10	3.2	4	1.2	1	0.5	—	—	0.003
*PTEN*	28	19	0.6	5	1.6	3	0.9	1	0.5	—	—	0.28
*SMAD4*	7	4	0.1	2	0.6	1	0.3	—	—	—	—	0.28
*STK11*	10	9	0.3	1	0.3	—	—	—	—	—	—	0.72
*TP53*	7	6	0.2	—	—	—	—	1	0.5	—	—	0.65

a A total of seven individuals had more than one VUS identified in the same gene, of which one individual had two VUSs in *PTEN*, one individual had three VUSs in *PMS2*, one individual had two VUSs in *MSH6*, two individuals had two VUSs in *CHEK2*, and two individuals had two VUSs in *MUTYH*.

**Table 3:** Characteristics of 10 individuals with early-onset colorectal cancer who identified as Black and had a VUS detected in *PMS2*

Sex	Age at CRC Diagnosis, years	Prior cancer history	*PMS2* VUS: nucleotide change^a^	*PMS2* VUS: sequence variation^b^	Gene and classification	Nucleotide change^a^	Sequence variation^b^
Female	42	Yes^c^	Ex13_Ex14del_PMS2CL?	Null	—	—	—
Female	25	No	Ex13_Ex14del_PMS2CL?	Null	—	—	—
Female	49	No	c.675A>C	p.E225D	—	—	—
Female	44	No	c.714C>A	p.S238R	—	—	—
Male	44	No	c.2179C>G	p.Q727E	—	—	—
Male	52	No	Ex13_Ex14del_PMS2CL?	Null	*CDH1*, VUS	c.1930G>A	p.D644N
Female	33	No	c.924G>C	p.E308D	—	—	—
Female	27	No	c.-7_-5DELTGC	Null	*MLH1*, VUS	c.1165C>T	p.R389W
Male	24	No	c.924G>C	p.E308D	*MSH6*, mutation	c.2150_2153DELTCAG	p.V717Afs*18
Female	53	No	c.506G>A	p.R169H	*MSH2*, mutation	c.1354G>T	p.E452*

Abbreviation: CRC, colorectal cancer.

^a^The “c” indicates that numbers begin with the first nucleotide of the cDNA.

^b^The “p” refers to the numbering of the amino acids in the protein.

c This patient had a first primary cancer diagnosis of lymphoma at 29 years of age.

Across other Lynch syndrome–related genes *MLH1*, *MSH2*, *MSH6*, and *EPCAM*, no overall differences were observed by self-identified race and ethnicity for the prevalence of VUSs (*P* ≥ 0.097; Table 2). However, the proportion of individuals with a VUS in *MSH2* statistically significantly differed between those who identified as White and as Asian (pairwise *P* = 0.01; Fig. 3D; Supplementary Table S7). Individuals with a VUS in *MSH2* who identified as Asian all had no prior cancer history and no clinically actionable PV/VLPs or other VUSs detected in any colorectal cancer susceptibility gene (Table 4). This pattern persisted in our adjusted models as individuals who identified as Asian were 3.14-fold more likely to present with a VUS in *MSH2* compared with those who identified as White (OR, 3.14; 95% CI, 1.17–8.45; *P* = 0.02; Fig. 3D; Supplementary Table S8).

**Table 4:** Characteristics of five individuals with early-onset colorectal cancer who identified as Asian and had a VUS detected in *MSH2*

Sex	Age at CRC Dx, years	Prior cancer history	*MSH2* VUS: nucleotide change^a^	*MSH2* VUS: sequence variation^b^
Male	45	No	c.-255C>A	Null
Male	33	No	c.1889G>T	p.G630V
Female	37	No	c.-145C>A	Null
Female	40	No	c.-255C>A	Null
Female	41	No	c.1145G>A	p.R382H

Abbreviation: CRC, colorectal cancer; Dx, diagnosis.

^a^The “c” indicates that numbers begin with the first nucleotide of the cDNA.

b The “p” refers to the numbering of the amino acids in the protein.

## Discussion

Our study focused on analyzing germline VUSs in colorectal cancer susceptibility genes for nearly 4,000 individuals diagnosed with a first primary early-onset colorectal cancer—of whom one in every four individuals identified as non-White. This work follows the recently updated National Comprehensive Cancer Network guidelines with universal genetic testing recommendations for patients with early-onset colorectal cancer that will undoubtedly drive significant advances in our ability to identify clinically actionable variants among all young patients and their families, increase surveillance for early detection, and support the discovery of curative therapeutic modalities. However, differences in accessibility to germline genetic testing will, in parallel, continue to exacerbate the pronounced racial and ethnic disparities that are known to exist within this patient population (2, 17–19). Recent findings from two Texas health systems by Dharwadkar and colleagues (20) shed an important light on this inequity, having found that individuals who identified as Black or Hispanic with an early-onset colorectal cancer diagnosis had significantly lower referral rates to genetic counseling and for individuals who identified as Black, the proportion of patients who also attended a counseling appointment was lower compared with the proportion of individuals who identified as White. Although there was initial evidence in this prior study that there may be differences in VUS rates by race and ethnicity, this cohort of 141 individuals with early-onset colorectal cancer who received germline testing across various panels did not have the benefit to comprehensively explore VUS patterns by race and ethnicity. Herein, we focused our study on comparing VUSs among individuals diagnosed with colorectal cancer at younger than 50 years of age who identified as White, Black, Hispanic, Asian, and Ashkenazi Jewish, with uniform clinical panel testing results for 14 colorectal cancer susceptibility genes.

It has been previously shown that among all individuals who undergo germline multigene panel testing, the overall VUS rates vary by self-identified race and ethnicity (8, 21, 22). This is partly attributable to the poor representation of diverse individuals in both population and clinical databases that are used for variant interpretation, which limits the ability to definitively classify variants as pathogenic or benign. Recent work in patients with colorectal cancer of all ages observed that overall rates of VUSs were lower for individuals who identified as Ashkenazi Jewish and higher for individuals who identified as Black and Hispanic (23). Similarly, in our early-onset colorectal cancer–specific cohort, we found that individuals who identified as Ashkenazi Jewish had the overall lowest VUS rates and individuals who identified as Black and Hispanic had the highest VUS rates, across all racial and ethnic groups. Even after accounting for the effects of patient age at colorectal cancer diagnosis, prior cancer history, sex, and status of a clinically actionable PV/VLP on these findings, we found that individuals who identified as Black and Hispanic retained significantly higher odds of presenting with a VUS in a colorectal cancer susceptibility gene compared with those who identified as White. Moreover, as all cases in our cohort underwent clinical multigene panel testing over 7 years ago, the persistence of these disparities in our cohort after sufficient time for variant reclassification points to long-term clinical care gaps by race and ethnicity in early-onset colorectal cancer. Notwithstanding that our results herein are consistent with this earlier work by Coughlin and colleagues (23), their variability in panel size and the number of genes (from 11–80+) tested as well as lack of information on age at colorectal cancer diagnosis shed light on unique contributions of our present study to the early-onset colorectal cancer literature.

Our study is novel in that beyond our observation of significant differences in overall VUS status by self-identified race and ethnicity specific to early-onset colorectal cancer cases, we also found that these patterns were largely driven by *PMS2* VUSs for individuals who identified as Black and *MSH2* VUSs for individuals who identified as Asian. This is particularly striking as clinically actionable variants in mismatch repair genes, including *PMS2* and *MSH2*, offer potential clinical implications for probands with early-onset colorectal cancer to support therapeutic decision-making (e.g., immunotherapy for localized and metastatic diseases) as well as for at-risk family members who may need to initiate disease surveillance at younger ages, at more frequent intervals, and at extracolonic sites. *PMS2* heterodimerizes with *MLH1* as a major component of the mismatch repair complex (24). Although PV/VLPs in *PMS2* are relatively rare in the etiology of Lynch syndrome, these are known to increase an individual’s risk of developing colorectal cancer compared with the general population (25). The identification and validation of *PMS2* germline variants has been a historical challenge (26) and is further complicated by the underrepresentation of individuals who identify as Black in reference databases for variant annotation as well as the few studies that have described germline genetic features specific to individuals who identify as Black. Earlier work by Guidalini and colleagues (27) found that two thirds of individuals who self-identified as being of African descent from the United States and harbored a PV/VLP in *PMS2* were diagnosed with a first colorectal cancer before 50 years of age. In South Africa, research-grade germline panel testing of 107 indigenous African individuals diagnosed with colorectal cancer at 60 years of age or younger identified and filtered three VUSs in *PMS2* (28). Included in these was the *PMS2* c.924G>C missense variant, which we discovered herein to comprise 20% of our *PMS2* VUSs for individuals with early-onset colorectal cancer who identified as Black. With the disproportionate burden of colorectal cancer (and particularly early-onset colorectal cancer), specifically among those who identify as Black, with the variation in population histories and genetic diversity, the unique VUS as well as PV/VLP (4) patterns across racial and ethnic groups point to distinct germline variant spectra and the potential for the future discovery of novel ancestry-specific variants associated with early-onset colorectal cancer. Moreover, as patients with Lynch syndrome harbor an elevated risk for the development of second primary malignancies after a colorectal cancer diagnosis (29), this compels the need for further clinical and laboratory-based investigations into existing colorectal cancer susceptibility genes like *PMS2*—specific to and representative of all population groups—in order to reduce clinical uncertainty. Together, these efforts will also support the optimization of clinical multigene panel test designs to be representative of disease risk in diverse population groups and provide optimal and equitable care management for all patients with early-onset colorectal cancer. Our results also point to a benefit for future studies to compare the burden of germline VUSs across diverse population groups between early-onset and late-onset colorectal cancer cases.

Moving toward early-onset colorectal cancer health equity, it is crucial to acknowledge that self-identified race and ethnicity is a social construct. These defined groups used herein are less precise and broader when compared with genetic ancestry—a fixed characteristic of the genome. As a consequence, there may be variation in ancestry within population groups (e.g., individuals who identify as Hispanic or Latino are an admixed group of European, Native American, and African ancestries; ref. 30) that we were unable to assess in our clinical cohort with uniform clinical sequencing data across 14 colorectal cancer susceptibility genes. To reduce some ambiguity, we limited our study to individuals who identified with only one racial and ethnic group. However, we do not yet know about germline variant patterns among individuals with early-onset colorectal cancer who identify as Alaska Native, American Indian, Middle Eastern, North African, or Pacific Islander or how these patterns fare in larger multigene testing panels. As we begin to disentangle this complex interplay of genetics with biology (31), social determinants of health, behaviors, and exposures across the life course (32), there remains much to be learned about how each of these factors independently and interdependently drives carcinogenic processes in the colorectum for young individuals across all populations. Both clinical and laboratory-based approaches will be integral to mechanistically define these key drivers underpinning early-onset colorectal cancer disparities, with downstream discoveries poised to shape this field and translate into actionable cancer prevention and interception, early detection, and even therapeutic strategies.

In conclusion, the findings from this study of VUSs among diverse individuals diagnosed with early-onset colorectal cancer revealed differing patterns of VUSs in variant reclassification and colorectal cancer susceptibility genes across racial and ethnic groups, even after variant reclassification, and yield key clinical implications. First, our results highlight the need for comprehensive genome-wide assessment of the germline variant spectrum and more diversity in the resources used for variant interpretation, particularly in populations who identify as non-White, and the importance of reclassification of VUSs resulting from these data—as this may alter clinical management. The potential for VUS reclassification over time also emphasizes the need to adapt genetic counseling strategies in the care setting, specifically for patients with a higher likelihood to have VUSs. This includes an emphasis on education about these variants with genetic testing given the hereditary cancer implications of genetic testing for young patients and their families. Collectively, these efforts will reduce uncertainty, eliminate inequities in the design of clinical multigene panel tests, and ultimately improve clinical management for a growing, diverse patient population.

## Supplementary Material

Supplementary Data Tables S1-S8
