# Supplementary material for: Variant of Uncertain Significance Patterns among Patients with Early-Onset Colorectal Cancer
Source: Cancer Res Commun. 2025 Feb 13;5(2):309–17. doi: 10.1158/2767-9764.CRC-24-0368 (PMC11824576; doi:10.1158/2767-9764.CRC-24-0368)
Supplement: Supplementary Data — Tables S1-S8 [file crc-24-0368_supplementary_data_suppst1-st8.docx]

*Supplementary Data*

**Variant of uncertain significance (VUS) patterns among patients with early-onset colorectal cancer**

Rachel A. Francis^1^; Sean V. Tavtigian^2^; Carolyn Horton^4^; Andreana N. Holowatyj^1,3,5-6^*

**Author Affiliations:**

^1^Department of Medicine, Vanderbilt University Medical Center, Nashville, TN

^2^Department of Oncological Sciences, ^3^Department of Population Health Sciences, University of Utah School of Medicine, Salt Lake City, UT

^4^Department of Clinical Diagnostics, Ambry Genetics, Aliso Viejo, CA

^5^Vanderbilt-Ingram Cancer Center, Nashville, TN

^6^Vanderbilt University School of Medicine, Nashville, TN

*Corresponding Author

**Table S1**. Prevalence and spectrum of 388 variants of uncertain significance (VUSs) in fourteen colorectal cancer susceptibility genes identified among 356 individuals with early-onset colorectal cancer.

|  | **Variants of uncertain significance (VUS)** | |
| --- | --- | --- |
|  | No. | % |
| Total VUSs | 388 |  |
| Cancer Susceptibility Gene |  |  |
| *APC* | 55 | 14.2 |
| *BMPR1A* | 12 | 3.1 |
| *CDH1* | 16 | 4.1 |
| *CHEK2* | 56 | 14.4 |
| *EPCAM* | 1 | 0.3 |
| *MLH1* | 36 | 9.3 |
| *MSH2* | 31 | 8.0 |
| *MSH6* | 58 | 14.9 |
| *MUTYH* | 25 | 6.4 |
| *PMS2* | 45 | 11.6 |
| *PTEN* | 29 | 7.5 |
| *SMAD4* | 7 | 1.8 |
| *STK11* | 10 | 2.6 |
| *TP53* | 7 | 1.8 |

**Table S2**. Frequency of variants of uncertain significance (VUSs) in colorectal cancer susceptibility genes by race and ethnicity among 356 patients who presented with at least one VUS.

|  |  | |  | **Race and Ethnicity** | | | | | | | | | | | | | | | |
| --- | --- | --- | --- | --- | --- | --- | --- | --- | --- | --- | --- | --- | --- | --- | --- | --- | --- | --- | --- |
|  | Study Population | |  | White | |  | Black | |  | Hispanic | |  | Asian | |  | Ashkenazi Jewish | |  | *P* |
|  | No. | % |  | No. | % |  | No. | % |  | No. | % |  | No. | % |  | No. | % |  |  |
| Total Patients with a VUS | 356 |  |  | 249 |  |  | 39 |  |  | 40 |  |  | 23 |  |  | 5 |  |  |  |
| Number of VUSs/Patient |  |  |  |  |  |  |  |  |  |  |  |  |  |  |  |  |  |  | 0.06 |
| 1 | 325 | 91.3 |  | 229 | 92.0 |  | 35 | 89.7 |  | 35 | 87.5 |  | 21 | 91.3 |  | 5 | 100.0 |  |  |
| 2-3* | 31 | 8.7 |  | 20 | 8.0 |  | 4 | 10.3 |  | 5 | 12.5 |  | 2 | 8.7 |  | 0 | 0.0 |  |  |

*A total of 30 patients in this cohort presented with 2 VUSs; one patient [who identified as White] presented with 3 VUSs.

**Table S3**. Summary of pairwise *P*-values between races and ethnicities by status of a variant of uncertain significance (VUS) in a cancer susceptibility gene. Cancer susceptibility genes include *APC, BMPR1A, CDH1, CHEK2, EPCAM, MLH1, MSH2, MSH6, MUTYH, PMS2, PTEN, SMAD4, STK11,* and *TP53*. Shaded boxes indicate *P*<0.05.

|  | **Variants of uncertain significance (VUS) status** | | | | |  |
| --- | --- | --- | --- | --- | --- | --- |
|  | White | Black | Hispanic | Asian | Ashkenazi Jewish | |
| White | -- | -- | -- | -- | -- | |
| Black | 0.01 | -- | -- | -- | -- | |
| Hispanic | 0.04 | 0.72 | -- | -- | -- | |
| Asian | 0.28 | 0.46 | 0.67 | -- | -- | |
| Ashkenazi Jewish | 0.08 | 0.007 | 0.01 | 0.03 | -- | |

**Table S4**. Odds of a variant of uncertain significance (VUS) among individuals with early-onset colorectal cancer.

|  | **Adjusted Model*** | | | | | | | | | | | | | | |  |  |
| --- | --- | --- | --- | --- | --- | --- | --- | --- | --- | --- | --- | --- | --- | --- | --- | --- | --- |
|  | Black vs. White | |  | Hispanic vs. White | |  | | Asian vs. White | | |  | | Ashkenazi Jewish  vs. White | | | |  |
|  | OR (95% CI) | *P* |  | OR (95% CI) | *P* | |  | | OR (95% CI) | *P* | |  | | OR (95% CI) | *P* | | |
| Any variant of uncertain significance (VUS)** | 1.55 (1.08-2.22) | 0.017 |  | 1.46 (1.02-2.08) | 0.037 | |  | | 1.28 (0.81-2.01) | 0.29 | |  | | 0.45 (0.18-1.10) | 0.08 | | |

Abbreviations: OR, odds ratio; CI, confidence interval; CRC, colorectal cancer.

*Adjusted for age at CRC diagnosis (years, continuous), sex at birth, prior cancer history (yes/no), and status of a clinically-actionable variant detected (yes/no).

**Any variants of uncertain significance (VUSs) in the following colorectal cancer susceptibility genes: *APC, BMPR1A, CDH1, CHEK2, EPCAM, MLH1, MSH2, MSH6, MUTYH, PMS2, PTEN, SMAD4, STK11, and TP53*.

**Table S5**. Summary of pairwise *P*-values between races and ethnicities by status of a variant of uncertain significance (VUS) in *PMS2*.

|  | **Variants of uncertain significance (VUS) status in *PMS2*** | | | | |  |
| --- | --- | --- | --- | --- | --- | --- |
|  | White | Black | Hispanic | Asian | Ashkenazi Jewish | |
| White | -- | -- | -- | -- | -- | |
| Black | 0.0004 | -- | -- | -- | -- | |
| Hispanic | 0.69 | 0.07 | -- | -- | -- | |
| Asian | 0.46 | 0.03 | 0.38 | -- | -- | |
| Ashkenazi Jewish | 0.27 | 0.04 | 0.22 | 0.45 | -- | |

**Table S6**. Odds of a variant of uncertain significance (VUS) in *PMS2* among individuals with early-onset colorectal cancer.

|  | **Adjusted Model*** | | | | | | | | | | | | | | |  |  |
| --- | --- | --- | --- | --- | --- | --- | --- | --- | --- | --- | --- | --- | --- | --- | --- | --- | --- |
|  | Black vs. White | |  | Hispanic vs. White | |  | | Asian vs. White | | |  | | Ashkenazi Jewish  vs. White | | | |  |
|  | OR (95% CI) | *P* |  | OR (95% CI) | *P* | |  | | OR (95% CI) | *P* | |  | | OR (95% CI) | *P* | | |
| Variant of uncertain significance (VUS) in *PMS2* | 3.59 (1.73-7.48) | 0.0006 |  | 1.24 (0.43-3.56) | 0.69 | |  | | 0.49 (0.07-3.65) | 0.49 | |  | | -- | -- | | |

Abbreviations: OR, odds ratio; CI, confidence interval; CRC, colorectal cancer.

*Adjusted for age at CRC diagnosis (years, continuous), sex at birth, prior cancer history (yes/no), and status of a clinically-actionable variant detected (yes/no).

**Table S7**. Summary of pairwise *P*-values between races and ethnicities by status of a variant of uncertain significance (VUS) in *MSH2*.

|  | **Variants of uncertain significance (VUS) status in *MSH2*** | | | | |  |
| --- | --- | --- | --- | --- | --- | --- |
|  | White | Black | Hispanic | Asian | Ashkenazi Jewish | |
| White | -- | -- | -- | -- | -- | |
| Black | 0.61 | -- | -- | -- | -- | |
| Hispanic | 0.79 | 0.57 | -- | -- | -- | |
| Asian | 0.01 | 0.22 | 0.08 | -- | -- | |
| Ashkenazi Jewish | 0.34 | 0.27 | 0.39 | 0.09 | -- | |

**Table S8**. Odds of a variant of uncertain significance (VUS) in *MSH2* among individuals with early-onset colorectal cancer.

|  | **Adjusted Model*** | | | | | | | | | | | | | | |  |  |
| --- | --- | --- | --- | --- | --- | --- | --- | --- | --- | --- | --- | --- | --- | --- | --- | --- | --- |
|  | Black vs. White | |  | Hispanic vs. White | |  | | Asian vs. White | | |  | | Ashkenazi Jewish  vs. White** | | | |  |
|  | OR (95% CI) | *P* |  | OR (95% CI) | *P* | |  | | OR (95% CI) | *P* | |  | | OR (95% CI) | *P* | | |
| Variant of uncertain significance (VUS) in *MSH2* | 1.36 (0.40-4.59) | 0.62 |  | 0.84 (0.20-3.60) | 0.81 | |  | | 3.14 (1.17-8.45) | 0.02 | |  | | -- | -- | | |

Abbreviations: OR, odds ratio; CI, confidence interval; CRC, colorectal cancer.

*Adjusted for age at CRC diagnosis (years, continuous), sex at birth, prior cancer history (yes/no), and status of a clinically-actionable variant detected (yes/no).

**There were no individuals who identified as Ashkenazi Jewish with a VUS in *MSH2* in this cohort.
